# Mixed Methods Evaluation of “Cooking Monsters”: An Empowerment-Focussed Aboriginal and Torres Strait Islander Adolescent Nutrition Programme

**DOI:** 10.1177/21501319261419935

**Published:** 2026-02-26

**Authors:** Renae Earle, Floyd Leedie, Robyn Littlewood, Simone Nalatu, Salifu Yusif, Jacqueline L. Walker

**Affiliations:** 1 The University of Queensland, St Lucia, Australia; 2 Goondir Health Services, Dalby, Australia; 3 Health and Wellbeing Queensland, Brisbane, Australia

**Keywords:** adolescents, obesity, prevention, mixed methods, programme evaluation

## Abstract

**Background:**

The Aboriginal and Torres Strait Islander Community Controlled Health Sector are well placed to address health equity concerns, including the disproportionate rates of obesity in Aboriginal and Torres Strait Islander adolescents. Building on previous research, this study piloted a co-designed empowerment-focussed obesity prevention programme with rural Aboriginal and Torres Strait Islander adolescents.

**Methods:**

Using mixed-methods, this research assessed nutrition and weight-related outcomes and explored programme acceptability and potential benefits through qualitative yarning. Seventeen adolescents participated.

**Results:**

There were no significant changes in quantitative weight-related and nutrition behaviour outcomes. However, qualitative insights suggest the programme was highly valued and viewed as a success. Community believed they could see observable changes in adolescent food literacy, empowerment and cooking confidence. Improvements for future delivery of the programme were identified by end-users.

**Discussion:**

This study is strengthened by implementing critical success factors for obesity prevention programmes and demonstrating ethical research practice with Aboriginal and Torres Strait Islander peoples.

**Conclusion:**

Future research opportunities continue to be actively explored by local community-controlled partners.

## Introduction

The Aboriginal and Torres Strait Islander Community Controlled (hereafter community-controlled) Health Sector are leaders in healthcare. Community-controlled services are grounded in culture and equity^1,2^ and deliver meaningful outcomes across Australia.^3,4^ Many community-controlled health organisations have established innovative models of care and community-led programmes that respond to the social and cultural determinants of health.^3-5^ This type of leadership is integral in prevention and management of overweight and obesity, particularly in childhood and adolescence.^6,7^

Overweight and obesity in childhood and adolescence is a public health priority in Australia and globally.^6,8,9^ Unhealthy weight in childhood and adolescence can negatively influence quality of life and impacts health outcomes later in life.^10-12^ Hence, programmes aiming to manage and prevent overweight and obesity, through targeting risk factors such as nutrition and physical activity, are of national interest.^6^ It is widely accepted that such programmes are more successful when co-designed with, and tailored to the needs of, the communities they aim to serve.^7,13,14^

The voices of young people are being increasingly recognised as critical in influencing paediatric health programmes and policy.^15-17^ Adolescence is a critical life stage, marked by rapid physiological and psychosocial development.^18^ Investments in adolescent health, including the prevention of obesity, can influence outcomes into the future and across generations.^18,19^ However, young people need to be involved in the design and delivery of these investments for them to succeed.^15-17^

This study builds on previous research that co-designed an obesity prevention programme with rural Aboriginal and Torres Strait Islander adolescents, and their community.^20^ Community-identified obesity prevention priorities informed the development of an empowerment-focussed nutrition programme to be situated within an after school social and emotional wellbeing programme delivered by the local community-controlled health service (hereafter, SEWB programme).^20^ The resultant programme, “Cooking Monsters” (hereafter, CM) is aligned with local community and health service needs and was implemented on the request of the health service.

The aims of this study were to:

1. Implement CM,^20^
2. assess the pre- versus post-programme impact on weight-related and nutrition behaviour outcomes and,
3. explore adolescent and community stakeholder experiences of the programme, including implementation barriers and enablers, and identify opportunities for future improvement.

## Methods

This study was quasi-experimental (pre- vs post-programme) and utilised mixed methods. Mixed methods were employed for the purpose of achieving a more complete understanding of the programme’s impact and implementation and to identify future recommendations. A mixed methods design acknowledges the complexity of health service research by measuring quantitative outcomes, and understanding, contextualising and building upon these quantitative findings through giving voice to the lived experience of participants.^21^

Research methods were developed iteratively with community and decision-making was shared with the local health service. Local Aboriginal colleagues advised on the cultural appropriateness of all methods, and shared control over the research process. This study was approved by The University of Queensland Human Research Ethics Committee A (2021/HE001531) and conducted with permission and support of the local community-controlled health service.

### Positionality Statement

This study was undertaken as part of the PhD candidature of the lead author (RE). She is a non-Indigenous woman and dietitian who lived and worked in the community where the research was conducted. RE established strong community relationships and aimed to centre Aboriginal and Torres Strait Islander perspectives, priorities and knowledge throughout the research. She has worked towards this by continuously reflecting on power dynamics, examining her biases and sharing leadership and opportunities with Aboriginal and Torres Strait Islander colleagues. RE has been guided by Aboriginal and Torres Strait Islander cultural mentors across all stages of the research and has aimed to co-create knowledge that reflects both Aboriginal and Torres Strait Islander and non-Indigenous epistemologies.

The wider authorship team bring diverse identities and experiences to this research. FL is a Wakka Wakka man and respected leader in the community-controlled health sector. The remaining authors (RL, SN, SY and JW) identify as non-Indigenous from various Australian and international backgrounds. We acknowledge that our diverse positionalities exist along an insider–outsider spectrum and influence the research.

This research was guided by decolonial and Indigenous Standpoint theories and re-Indigenising research frameworks.^22-25^ Governance and decision-making were shared with the local community-controlled health service who co-led the project. The research was conducted in accordance with community-control and ethical research principles.^1,2,26^ Findings have been returned to community-controlled partners through appropriate and reciprocal means.

### Research Setting

This research was situated within a rural Queensland (Australia) community, in which one-third (29.7%) of residents identify as Aboriginal and/or Torres Strait Islander.^27^ It was undertaken in partnership between the local community-controlled health service, The University of Queensland and Health and Wellbeing Queensland.^28^

Research was undertaken within the local health service, specifically, within an after-school SEWB programme for adolescents. This SEWB programme was highly valued amongst the community and perceived as generating positive outcomes for youth.^28^ It was identified by leaders of the local health service, and community members, as a suitable setting for this research.^20^

### Programme Description

CM was co-designed with adolescents and their community in an associated study.^20^ The programme was tailored to the implementation context and designed for delivery within the existing SEWB programme. It was delivered between October and December of 2021 and co-facilitated by the lead author (RE) and the local Aboriginal SEWB programme facilitator (hereafter, local co-facilitator). In the interest of preparing the local co-facilitator to deliver the programme on-going (after completion of the research project) a train-the-trainer model was employed.

### Participants and Recruitment

Adolescents, their parents/caregivers, community stakeholders and local health service staff were purposively recruited. Adolescents were recruited to participate in the programme and contribute pre- and post-programme data. Adult participants (parents/caregivers and health service staff) were invited to contribute to post-programme qualitative aspects of this research, only. Participants and recruitment methods used in this study are similar to previous research conducted in this setting.^20,28^

Adolescents were recruited to this research through their involvement in the existing SEWB programme. All participants of the SEWB programme were invited to participate in the CM programme (which took place during usual SEWB programme sessions). The lead researcher had established relationships with adolescents and the SEWB programme staff through previous collaborative research.^20,28^ She explained the optional research processes associated with CM to adolescents, who provided their voluntary, informed written consent. Adolescents were also required to obtain the written consent of their parent/caregivers to participate in research processes. The lead author supported adolescents and their families to understand and consent to the research, whilst ensuring there would be no negative consequences if they chose not to participate.

Adult stakeholders who were involved with the SEWB and CM programmes (parents/caregivers, health service staff and community leaders) were invited to participate in post-programme qualitative research. Adult participants were purposively recruited based on their involvement with the programme. All adult participants provided their voluntary, informed written consent.

### Data Collection

Data collection occurred at the usual time and place of SEWB programme sessions (after school within a community wellbeing centre). Data collection activities were conducted by the lead researcher and the local co-facilitator, who shared control over the process. Pre- and post-programme data collection took place in the week preceding and proceeding programme delivery, respectively.

### Programme and Participant Characteristics

For all participants, demographic information related to age, gender and cultural identity was collected through a written survey form. At each CM programme session, adolescent attendance was recorded.

#### Quantitative Measures

Data regarding adolescent height, weight and self-reported nutrition behaviours were collected pre- and post-programme. Collecting information related to health outcomes was already embedded within SEWB programme processes. Therefore, data collection activities and associated processes conducted as part of this research were not unfamiliar to participating adolescents and the local co-facilitator.

Under supervision of the local co-facilitator, adolescent height and weight were measured by the lead researcher with a Seca stadiometer and analogue body weight scale. Standardised procedures were followed.^29,30^ Each procedure was performed twice, and the average of the 2 measurements was recorded. From height and weight measurements, BMI, BMI percentile (%-BMI) and BMI z-scores (z-BMI) were calculated using an electronic growth chart tool.^31^ Where data about a participant’s age was missing, it was substituted with the median age of all participants in order to calculate %-BMI and z-BMI. This was deemed appropriate as the age range of all participants was narrow and this assumption allowed for more thorough interrogation of the data. All BMI measures were interpreted using internationally accepted standards for classifying weight status in adolescents.^32^

To assess nutrition behaviours, adolescents were asked to complete an adapted Short Form Food Frequency Questionnaire (SF-FFQ).^33^ This tool was selected because SF-FFQs have demonstrated good reliability and validity with Aboriginal and Torres Strait Islander youth^34-36^ and it was deemed appropriate by the local co-facilitator. The SF-FFQ collects information about the frequency of consumption of various core and discretionary food groups over the previous month. The SF-FFQ contains:

- Thirteen items about the consumption of non-meat foods answered on an 8-point Likert scale,
- Seven items about the consumption of meat (including fish) answered on a 6-point Likert Scale and
- Two items about daily consumption of fruit and vegetables serves answered as free text.

#### Qualitative Data

Qualitative methods were employed post-programme to understand participant perceptions of, and experiences with, the CM programme. Qualitative data collection took place in December 2021 with both adolescent and adult participants. In alignment with Indigenous Standpoint and De-Colonial theories, the Aboriginal and Torres Strait Islander research method of yarning was used with the support of the local co-facilitator. A yarning circle is a collaborative discussion enabling the sharing of information whilst building respect and trust.^25,37,38^ Data collection procedures related to yarning circles are outlined in associated studies.^20,28^ Yarning circles were co-facilitated by the lead researcher and the local co-facilitator. The local co-facilitator cross-checked and approved yarning questions which explored topics related to programme content, delivery, facilitation, evaluation, on-going implementation and quality improvement.

Adult participants were offered the option to contribute to the research via yarning circle or a written qualitative survey. The survey contained questions similar to those covered in yarning and were designed as a lower participant burden option.^20^

### Data Analysis

#### Programme and Participant Characteristics

Descriptive statistical analyses were performed on programme and participant characteristic data with the assistance of Microsoft Excel 2025.^39^

#### Quantitative Measures

Data were assessed for normality through visualisation and the Shapiro-Wilk test, performed with the assistance of GraphPad Prism 2024.^40^ Outliers were identified (through visualisation) and discussed with the research team to reach a collective decision on their inclusion. Non-matched pre-/post-programme data were excluded from pre- versus post- analyses. For all tests, statistical power was set at 0.8 and a *P* value <.05 was considered significant. All tests were 2-tailed.

Pre- versus post-programme data comparisons related to BMI (BMI, %-BMI and z-BMI) were performed using Wilcoxon Signed-Rank test, with the assistance of GraphPad Prism 2024.^40^ For nutrition behaviour (SF-FFQ) data, pre- versus post-programme comparisons were tested for each item individually, before being grouped into like items for further analyses (eg, salad and cooked vegetable items were subsequently analysed as 1 “vegetables” group). Each food group was then tested for significant changes using the same statistical analyses. For further information on SF-FFQ item grouping, please see Supplemental File.

Data about daily serves of fruit and vegetables were interpreted with respect to relevant Australian Dietary Guidelines (ADGs) to determine the proportion of adolescents meeting/not meeting national recommendations.^41^ Data were converted to a 2 × 2 contingency table and tested for pre- versus post-programme differences using McNemars test, with the assistance of GraphPad Quick Calcs.^42^ This software was also used to calculate a pre- versus post-programme Odds Ratio. Standard guidelines about interpreting Odds Ratios were utilised.^43^

#### Qualitative

Audio recordings were transcribed verbatim with the assistance of Rev Artificial Intelligence (AI) transcription services.^44^ Rev AI operates in accordance with privacy laws and a Data Processing Policy.^44^ These regulations ensure the security, confidentiality and integrity of data and does not risk exposure of the data to humans outside the research team. The use of AI is growing in qualitative research fields and offers potential benefits when risks are appropriately managed.^45-47^

Inductive thematic analysis was used to identify pertinent information emerging from qualitative data. Data analysis was performed by the lead author, with the assistance of QSR NVivo 11.^48^ To further interrogate the data, emergent themes were positioned within the integrated Promoting Action on Research Implementation in Health Services (i-PARIHS) framework.^49,50^ This framework was selected as it has been designed for use in the health service setting and utilised in similar research contexts.^20,51-53^

Quantitative and qualitative results are reported separately in this study. However, both data sets were considered holistically to generate integrated discussion points that identify the overall strengths, weakness and recommendations of this mixed methods research.

## Results

### Programme and Participant Characteristics

This research sought to deliver the CM programme with the highest possible level of intervention fidelity.^20^ Mostly, specific programme elements and delivery mechanisms that were strongly favoured by the community in previous research were retained.^20^ In some cases, programme facilitators were required to deviate from the CM programme outline by altering and/or omitting particular sessions. These deviations occurred in response to community needs and/or the implementation context. For example, community events, weather events, Sorry Business and the availability of local health service staff and other community partners. In other instances, minor content changes were made in response to real time feedback from adolescents and the local co-facilitator.

A description of the programme delivered, and session attendance, is summarised in the Supplemental File. In total, the programme delivered 9 sessions over 6 weeks.^20^ Programme attendance remained relatively stable over time and was strongly influenced by attendance at the SEWB programme. For example, weeks during which there were fewer attendees as the SEWB programme, there were also fewer attendees of the CM programme.

In total, 23 adolescents participated in the CM programme. Seventeen of these adolescents provided their (and parent/caregiver) consent to participate in research processes. The median age of adolescent participants was 13.5 years (range = 12-16 years; reported by n = 11). Approximately half (59%, n = 10) of participants were female and most (95%, n = 16) identified as Aboriginal. On average, each participant attended 60% of CM programme sessions (equivalent to 5.4 sessions per participant).

### Quantitative Pre- Versus Post-Programme Measures

One outlier was detected, who, was suspected to have Prader-Willi Syndrome upon discussion with local health service staff. This participant had a higher BMI than their peers, however, on a small number of items, they self-reported healthier nutrition behaviours on the SF-FFQ compared to their peers. Based on the observations of those involved in data collection, the outlier was assessed as a true value. Pre- versus post-programme analyses were repeated excluding the outlier and results were not significantly changed. For these reasons, the outlier was included in the following analyses.

Twelve adolescents contributed pre-programme anthropometric data. Of these 12, 83% (n = 10) had a BMI in the normal range and 17% (n = 2) had a BMI in the obese range. Post-programme, 7 adolescents contributed anthropometric data. Of whom, 71% (n = 5) had a BMI in the normal range and 29% (n = 2) had a BMI in the obese range. These figures roughly approximate what would be expected for Queensland Aboriginal and Torres Strait Islander children aged 2 to 17 years.^54^ Data analysis on 6 pre- versus post-programme matched pair comparisons indicated there were no significant changes in any BMI metric. See Table 1.

**Table 1.** Weight-Realted Outcomes Pre- vs Post-Programme Comparison.

Metric	Pre-programme^a^ median (range)	Post-programme^b^ median (range)	Median of pre- vs post-programme difference^c^ (confidence interval)	W value (sum of signed ranks)	*P* value
BMI (kg/m^2^)	20.24 (15.49, 42.10)	20.72 (16.98, 39.96)	−0.04 (2.14, 0.91)	−5	.69
BMI z-score	0.41 (–1.63, 3.05)	0.54 (–0.71, 2.87)	−0.03 (−0.55, 0.09)	−9	.44
BMI percentile	65.97 (5.17, 99.89)	70.58 (23.91, 99.73)	0.24 (−20.33, 2.47)	−3	.84

a n = 12.

b n = 7.

c n = 6.

Fifteen adolescents completed the nutrition behaviour SF-FFQ survey pre-programme and 8 completed this measure post-programme (7 were pre-/post-programme matched pairs). Amongst individual SF-FFQ items, there were no statistically significant differences pre- versus post-programme for matched pairs. See Supplemental File.

Following preliminary testing of individual SF-FFQ items, similar food/drink items were groups for further analysis. Pre- versus post-programme grouped item analyses were also not significant, except for unprocessed, processed and total meat/fish. See Table 2. Pre- versus post-programme differences in these groups were small but statistically significant. Meat consumption trended downwards (total meat pre-programme median = 4 and post-programme median = 2, unprocessed meat pre-programme median = 4 and post programme median = 2 and processed meat pre-programme median = 4 and post programme median = 3). When analyses were repeated excluding the outlier, pre- versus post-programme differences in processed meat/fish were no longer significant (*P* = .635), while significant changes in unprocessed and total meat/fish consumption remained (*P* < .001 for both groups, still trending towards reduced consumption post-programme). This suggests that in this small sample size, trends towards reduced processed meat consumption may be driven by the behaviours of 1 outlier.

**Table 2.** Change in Nutrition Behaviour Pre- vs Post-Programme for Food/Drink Groups (measured by a Food Frequency Questionnaire; n = 8).

Food/drink group	Median of pre- vs post- programme differences (confidence interval)	W value (sum of signed ranks)	*P* value
Combined fruit	0 (0, 1)	21	.29
Combined vegetables	0 (0, 1)	6	.70
Total fruit and vegetables	0 (0, 1)	51	.23
Fiber-rich Carbohydrates	0 (−2, 1)	−26	.27
Discretionary food	0 (−2, 1)	−67	.35
Unprocessed meat and fish	0 (−1, 0)	−141	<.01*
Processed meat and fish	0 (−1, 0)	−62	.02
Total meat and fish	0 (−1, 0)	−377	<.01
Total serves of fruit and vegetables	0 (−1, 1)	3	.86

* *P* not <.05 in repeated analyses with the exclusion of n = 1 outlier.

A significant proportion of adolescents participating in this research did not meet the ADGs for daily fruit and vegetable consumption. This did not significantly change pre- versus post-programme but trended towards improvement post-programme. See Table 3.

**Table 3.** Pre- vs Post-Programme Changes in the Proportion of Adolescents Meeting the Australian Dietary Guidelines for Daily Fruit and Vegetable Consumption.

	% meeting ADG		Matched pair pre- vs post-programme comparison (n = 8)	
Nutrition behaviour	Pre-programme (n = 14)	Post-programme (n = 8)	Odds ratio (confidence interval)	*P* value
Daily fruit consumption	43 (n = 6)	63 (n = 5)	1 (0.01, 78.50)	.48
Daily vegetable consumption	7 (n = 1)	13 (n = 1)	1 (0.01, 78.50)	.44

### Qualitative Results

Two yarning sessions were conducted: 1 with adolescents (n = 6), and 1 with health service staff (n = 1). One parent/caregiver and 1 community leader opted to contribute qualitative data via written survey. The health service staff participant was the local co-facilitator.

Most (78%) participants providing qualitative data identified as Aboriginal. All adolescents participating in yarning identified as Aboriginal. The median age of adolescents participating in yarning was the same as the overall sample (13.5years). However, there were a higher proportion of girls participating in yarning compared to the overall sample (67% identified as female, compared to 59% in the overall sample).

From thematic analysis, 2 themes emerged. An additional 5 key messages were also identified which, despite being driven largely by the response of 1 participant, capture important findings relevant to this research. Overall, themes and messages suggest the programme was highly valued by the community and benefited adolescents. Participants viewed the CM pilot programme as a learning opportunity and discussed opportunities for improvement. Themes, key messages and related verbatim participant quotes are described below. Further participant quotes are included in the Supplemental File.

Overall, themes and key messages demonstrated alignment to several aspects of the i-PARIHS framework. Themes and key messages related strongly to the innovation and recipients dimensions of the i-PARIHS. To a lesser degree, connections to the facilitation and local context dimensions were also identified.

#### **Theme 1**: The Cooking Monsters Programme Was Valued by Community

Yarning with all stakeholder groups indicated the programme was highly valued by the community and perceived as delivering meaningful benefits for adolescents. In the words of a participating parent, “*I was happy with my small experience as I got to taste some good food and it was great to see the excitement in the kids when they produced their food for us*.” Amongst adolescents, the programme was seen as fun, engaging and educational. All participants valued the programme’s focus on health and cooking skills. One adolescent commented, “*Cooking was Amazing. Yeah. Was Healthy!*”. Participants commented about adolescent gains in nutrition and cooking knowledge, confidence and engagement. These benefits contributed to participants’ satisfaction with the programme and local pride. One community leader commented, “*I think the program is something to be proud of. Whenever I have spoken to those outside of the community about the program, they are always impressed that the community has these types of initiatives.*”

#### **Theme 2**: Observable Results Related to Food Literacy and Empowerment

All participants commented on the benefits of the programme for adolescents. Perceived benefits included outcomes related to cooking and nutrition knowledge/skills, overall confidence, empowerment and social and emotional wellbeing. Adolescents mostly commented on what they had learned, rather than how they felt (eg, improved confidence). For example, 1 adolescent commented, “*How we learn how to like chop up stuff and be careful (in the) kitchen. Be careful with the knife*.” While adolescents agreed they felt more confident through their experiences with the programme, wellbeing benefits were more strongly raised by adult participants. For example, in the words of a community leader, “*I think there has been a change as kids have been getting excited to be able to prepare these meals or even talk about meals they are going to cook away from the program*.” Other participants also speculated that the programme was having a potential impact at the household level by encouraging cooking practices at home. For example, 1 parent commented, “*Some of the kids are talking about cooking at home*.”

#### **Key Message 1**: The Programme Became More Acceptable Over Time

This key message was most strongly raised by the local co-facilitator. It was also commented on by a community leader, but to a lesser extent. The local co-facilitator expressed initial reservations about CM and its degree of fit within the SEWB programme. They commented that they were unsure of how adolescents would receive CM, as it was different to their typical experience of the SEWB programme. Over time, as the local co-facilitator and adolescents were exposed to the programme, these concerns faded. By the end of the programme, the local co-facilitator could clearly articulate the benefits of CM and noticed the enjoyment of participating adolescents. This is demonstrated in the following quotes from the local co-facilitator; “*I think they (adolescents) thought it was gonna take away from what they do with (the SEWB program). Yeah. Um, but you’ve seen on Murri MasterChef Day. Mm. How they, how much they were into it and how much pride they had in what they were doing. So much pride*,” and, *“I was sceptical at the start. I was like, I don’t know if these kids are wanting to cook. I think now that they’ve done it, and they got into it*.” Key elements of CM programme, such as being fun, interactive and voluntary contributed to it being viewed as a good fit for the SEWB programme. These reflections raised by the SEWB co-facilitator were not raised by adolescents who participated in this study.

#### **Key Message 2**: The Pilot Was a Successful Learning Opportunity and Future Recommendations Were Identified

The CM programme was delivered as a pilot to test its acceptability, feasibility, barriers and enablers. Adult participants (particularly the local co-facilitator) discussed the programme as a successful pilot because it sparked learnings that would improve its delivery in the future. For example, the local co-facilitator commented, “*I think it would be good to come back again and rerun the programme Yeah. With a couple of, um, you know, of the suggestions we made*.” Adult participants, particularly the local co-facilitator, noted the process of piloting and continuous quality improvement as consistent with the local health service culture of innovation and learning. Hence, they suggested that the research process was consistent with the usual ways of working within the implementation context. This was evidenced in the following quotation from the local programme coordinator “*Everything we do at (SEWB Program) is just the trial. Yeah. See how it goes. And reflect . . . Pretty much what we’re doing. Yeah. Try and better with something else that we do next time*.” Adolescents, however, did not comment on the programme as a trial or pilot, and posed fewer recommendations about future delivery.

Recommendations for improving CM are outlined below (key messages 3-5) and relate to the innovation, facilitation and resource domains of the i-PARIHS framework.

#### **Key Message 3**: Further Embedding Cooking Monsters Within the Local Implementation Environment Could Improve Engagement With Hard-to-Reach Adolescents

The local co-facilitator and parent/caregiver participants suggested that CM could be improved by further embedding itself within the local implementation environment. For example, 1 parent commented, “*It is something that could be moulded into other activities*.” It was suggested that further integrating CM and the SEWB programme would increase clarity about the programme, improve planning and help CM tap into existing adolescent motivation and engagement systems. For example, the SEWB programme operates on a points system which motivates adolescents to engage in SEWB activities within and outside of the programme. The local co-facilitator commented, “*It (CM) could be incorporated to the point system. Um, I’m just thinking of, of a couple of boys that had no interest in it. Mm. Um, I think it, I think also if it was something that was consistently put into the plan, Mm. Consistently week in, week out, um, I think they, that those guys would start to enjoy it. Especially if they, you know, like when we’re making our stuff for, um, for afternoon tea today, like Yeah*.” Integrating motivation strategies such as the points system was seen as particularly beneficial for adolescents who were less likely to engage in cooking activities (eg, boys who prefered to play games rather than cook). However, engaging adolescents in activities they might perceive as less fun was noted as a universally difficult challenge. The local co-facilitator commented, “*I also think, like, if you can get those guys that have no interest in it to actually cook something that they really like and they want to cook it. Yeah. And then they’re like very proud of it. Um, I think they’re inclined to do it again. Yeah. Um, yeah. That is a tricky one. Mm. I suppose if we had the answer to that, Then we’d have the answer and teaching would be easy at school*.”

#### **Key Message 4**: Overcoming Resource, Time and Equipment Constraints Could Improve Programme Delivery in the Future

While the local co-facilitator (and others) suggested several ideas to improve the CM programme, they acknowledged that the implementation of these ideas would require additional resources (time, personnel, funding and cooking infrastructure/equipment). The local co-facilitator indicated the need for adolescents to spend more time in the CM programme. They suggested the programme should run for a longer duration and/or be more intensive in its approach. They commented, “*I think just having, having more time with you (the lead researcher). Two days a week over an eight-week thing. Yeah. Or one day a week over a 10 week*.” and, “*then you’re here through the school holidays where you can, you know, (do) the two-week walkthrough of school holidays, you can really drum it up. Oh yeah. Because you’ve got ’em all day.*”

The local co-facilitator also raised the challenge of having many adolescents to teach and only 1 or 2 programme facilitators. In response, they suggested adolescents could be divided into groups to reduce the burden on facilitators and/or increase the number of cooking stations for adolescents (which would require the purchasing of additional equipment). This is evidenced in their own words, “*I’m just trying to work out a way how you can get through 20 kids or 25 kids or whatever it is, that are in an activity through cooking. Whether we split ’em into different groups, whether it’s five, and this group’s cooking this on Monday, this one’s cooking this on Tuesday so that all the others are occupied doing something (else), then you’ve just got five kids in there doing it (cooking).*” And, “*It’s just, um, the tricky thing is, like you said, it’s the amount of kids, um, having all the equipment that’s needed to make it successful so that every kid can, every kid can cook or do it in groups*.” While adolescents did not discuss many recommendations about how to improve the programme, they did echo the suggestions of the local co-facilitator by commenting on the need to create smaller cooking groups. Meanwhile, parents also suggested increasing the time and resources dedicated to the programme to maximise its benefits. One parent commented, “*I do think being happy with program helps a little bit, but it does need more than that. It needs interest and backing from organisations and time and effort from people who are involved*.”

#### **Key Message 5**: Relationships Are Central to Programme Success and Need to be Continued

Staff and community leader participants discussed the importance of strong relationships in successful programme delivery. They positioned the facilitator (and their relationships with adolescents and the broader community) as a key element of programme implementation. One community leader described the role of the programme facilitator in the following, “*This person is key to increasing (adolescent) willingness to participate in these programs and help connect the dots between their (adolescents’) experience and life lessons which is empowering*.” The local co-facilitator suggested that for anyone to be a successful facilitator, they needed to build strong relationships. To do this, it was suggested that they needed to be completely embedded in the programme. They commented, “*You really have to build a relationship and for you to build a relationship, you’ve gotta be around a little bit. Mm. And participating in everything . . . that they’re doing.*” The local programme co-ordinator was not favourable to someone new coming to facilitate future iterations of the project, because (in their words), “*they’re going to not do too much for the first month or whatever. It’ll be just about getting there, forming a (relationship).*” Recommendations about the facilitators were not made by adolescents participating in this research.

The Supplemental File contains additional verbatim participant quotes relevant to each of the themes and key messages.

## Discussion

Through implementing a co-designed Aboriginal and Torres Strait Islander adolescent nutrition program, this research has gathered new insights about obesity prevention in the community-controlled setting. This study joins a growing body of literature about obesity prevention programmes with Aboriginal and Torres Strait Islander young people.^55^ Outcomes indicate this programme had minimal impact on adolescent BMI outcomes and nutrition behaviours. However, themes emerging from qualitative research indicate adolescents and their broader community, were generally satisfied with the programme and viewed it as beneficial. Stakeholders suggested several recommendations to improve programme implementation, which are informing future endeavours.

The programme implemented through this research was co-designed with its recipients.^20^ The CM programme outline was informed by consultation and advice from local stakeholders.^20^ Due to various influences, the programme was unable to be delivered exactly as intended. The programme was implemented in Term 4 of the school year, during which, a myriad of school and community activities occupied adolescents, their families and the broader community. For example, school camp, Christmas celebrations, end-of-school year awards night and community sporting events. At the same time, the region in which this research was conducted was significantly impacted by flooding (specifically during the weeks of post-programme data collection). These factors, amongst others (such as the availability of local health service staff and community partners to support programme activities), impacted intervention fidelity. This was not unexpected – previous studies implementing obesity prevention programmes in the community setting have also experienced issues related to fidelity.^56^

The version of CM that was implemented in this research was 2 weeks shorter in duration than initially planned due to the factors outlined above.^20^ This was noticed by participants who described the need to extend the length of the programme, to maximise its impact. Participants suggested a more intensive programme delivery approach (eg, longer duration or longer sessions) to achieve a bigger CM programme “dose.” Across Australia, the length of obesity prevention programmes in Aboriginal and Torres Strait Islander youth is highly varied (from 7 weeks to greater than 12 months),^55,57-61^ and there is no clear established relationship between duration (or “dose”) and programme effectiveness.^62,63^

Due to competing demands on the time of local health service staff, the SEWB programme (and, by association, CM) was cancelled for 1 week. It was during this (cancelled) week that programme activities related to understanding and navigating the local food system were planned. Unfortunately, there were no opportunities (that were reasonable for the project timeframes) for this element of the programme to be rescheduled. This is significant because in co-designing CM, confidence to navigate the food system and make healthy choices was a key theme.^20^ Further, evidence suggests that incorporating food system knowledge into multi-faceted nutrition programmes can be beneficial.^64,65^ It is unfortunate that this important element of the programme was unable to be executed as part of this research. However, there are still ways in which the local food environment was recognised in programme delivery. For example, ingredients used in cooking recipes were familiar, affordable and accessible within the local environment and local food/drink outlets were generally discussed with adolescents.

Overall, this programme had little significant impact on quantitative outcomes. It is unsurprising that BMI outcomes did not change significantly, given the short duration of the programme and the various other factors that impact weight status.^8,66,67^ Our findings are consistent with current literature about Aboriginal and Torres Strait Islander childhood obesity programmes, which rarely demonstrate significant improvements in weight.^55,68^ A global study assessing the effectiveness of Indigenous childhood obesity prevention programmes found only 8.8% of studies significantly impacted a weight-related outcome, and these programmes were usually much greater than 6 weeks in duration.^55^ Regardless, researchers were encouraged to include weight as one of the outcomes in this study (despite its short duration as a pilot programme) by some members of the local community and the local health service. Further, the SEWB programme was already moving towards measuring BMI as part of its usual service delivery (prior to this study) for the purposes of monitoring this metric in its participants over the long term. Hence, despite it being unlikely to significantly change within this study, BMI was included as an outcome of interest.

Changes in other health outcomes, such as nutrition behaviours, however, are more common amongst other studies (51.9%)^55,68^ and could have been reasonably anticipated in this research. In Australia, several childhood obesity prevention studies have demonstrated improvements in nutrition behaviours.^55,68^ For example, improved self-report fruit and vegetable intake and reduced sugar sweetened beverage intake.^57-59,61^ However, the sample size of those studies was considerably larger than that of this study.

Fruit and vegetable intake is of particular interest, given the vast proportion of Australian children are not meeting the national recommendations.^54^ This research demonstrated promising (but not statistically significant) changes in the proportion of programme participants meeting fruit/vegetable guidelines. Post-programme, the proportion of participants meeting fruit guidelines was in line with the national average (national average = 57.1%, post-programme = 62.5% (up from 42.9% pre-programme)). Post-programme, the proportion of participants meeting vegetable guidelines exceeded the national average (national average = 6%, post-programme = 12.5% (up from 7.14% pre-programme)). However, these results should be interpreted with caution. Upon further interrogation of the data, it is suggested that observed changes are likely due to participants who were not meeting guidelines being lost to follow up, rather than the impacts of the CM programme.

### Strengths

Themes emerging from yarning circles indicate the programme was highly valued and accepted by the community. This was expected given the programme was designed for and with its recipients, responded directly to community needs and was approved by local Aboriginal stakeholders.^20^ This research reinforces the benefits of co-design and sharing leadership with Aboriginal and Torres Strait Islander communities.^69-72^

Despite minimal shifts in quantitative outcomes, qualitative insights suggest the community viewed the CM programme pilot as a success. This highlights the value of a mixed methods approach. In the absence of either qualitative and quantitative data sets, the findings, conclusions and future recommendations of this research would be incomplete.

Community suggested benefits related to nutrition knowledge/attitudes and adolescent confidence and empowerment were realised. While the sample size was small, there are other information sources that affirm the programme was valued by the community. For example, the local health service has showcased the research at community events, continues to engage with researchers to develop future programme implementation resources, and are actively pursuing funding opportunities to continue the CM programme. The partnership through which this research was undertaken remains strong.

### Limitations

Of the 17 adolescents who consented to participate in this research, far fewer contributed quantitative or qualitative data. Approximately 50% of participants were lost to follow up despite attendance remaining steady throughout the 6 weeks of programme delivery. This level of attrition is not uncommon amongst similar programmes (ranging from 27% to 73% lost to follow up).^73^ However, it did significantly impact sample size and the statistical power of this study. There are many factors impacting attrition in obesity prevention programmes.^73^ In this study, authors attribute the low post-programme participation rates to factors outside the control of researchers. For example, end of school year busyness and regional flooding impacted the availability of parents and their families to attend the SEWB programme and participate in post-programme data collection. Regardless, low sample size has limited this study, and results should be interpreted accordingly.

Qualitative feedback about the programme suggests adolescents did experience benefits from participating in the programme. Adolescents and their community commented that adolescent knowledge and attitudes towards cooking and nutrition had improved. However, these outcomes were not measured quantitatively in this research. Hence, highlighting a potential tension between quantitative and qualitative markers of programme success identified in this mixed method research.

Based on qualitative feedback, appropriate tools to measure food and nutrition literacy in Aboriginal and Torres Strait Islander adolescents may have been sensitive to change in this study. However, there is little consensus on a standardised and appropriate tool for measuring food and nutrition literacy in Aboriginal and Torres Strait Islander young people^74,75^ and further research in this field is required.

Themes from yarning circles suggest changes in adolescent confidence and empowerment were observed, through their exposure to the programme. In preliminary work, researchers tested a validated empowerment measurement tool with adolescent participants of this study (The Growth and Empowerment Measure Short Form, GEM-SF).^76,77^ However, it was not found reliable or valid in the study population.^78^ There is a need to adapt the GEM-SF to be appropriate for adolescents.^78,79^ An adapted adolescent GEM-SF would allow researchers and health professionals to accurately monitor and report on empowerment changes in relation to CM, and other relevant programmes/services.

### Implications and Recommendations

The are several opportunities identified by participants of this research that, if implemented, would contribute new knowledge about obesity prevention in Aboriginal and Torres Strait Islander adolescents. Recommendations about improving CM are expected to maximise benefits and reduce implementation barriers. These include increasing the duration and/or dose of the programme, further embedding the programme within the local health service and maintaining strong community-facilitator relationships. Further funding and resources to achieve this are required and are being actively pursued by the local health service.

Finally, there are various elements of this research approach that could be utilised by other research groups into the future. For example, co-design methods, community partnership and the integration of Aboriginal and Torres Strait Islander and implementation science frameworks. However, the CM programme content and delivery were designed specifically for the local community in which the research was situated and addresses their unique interests and needs. Further, the CM programme was tailored for delivery within the context of an after-school SEWB programme. Hence, without adaptation, CM may have limited transferability to other communities or settings. Continued research on place-based obesity prevention initiatives is encouraged.^6^

## Conclusion

Supporting the health of Aboriginal and Torres Strait Islander adolescents requires community partnership and local support. This research implemented a bespoke nutrition and empowerment-focussed obesity prevention programme that was co-designed with adolescents and their broader community. Community feedback suggests the programme was well accepted by adolescents and their community and delivered meaningful benefits. These benefits, however, were not reflected in quantitative measures related to BMI and nutrition behaviours. Partnerships established through this research remain strong. The local community-controlled health service (in which this research was situated) continues to pursue opportunities to implement the recommendations of this research and sustain programme delivery.

## Supplemental Material

sj-docx-1-jpc-10.1177_21501319261419935 – Supplemental material for Mixed Methods Evaluation of “Cooking Monsters”: An Empowerment-Focussed Aboriginal and Torres Strait Islander Adolescent Nutrition Programme Supplemental material, sj-docx-1-jpc-10.1177_21501319261419935 for Mixed Methods Evaluation of “Cooking Monsters”: An Empowerment-Focussed Aboriginal and Torres Strait Islander Adolescent Nutrition Programme by Renae Earle, Floyd Leedie, Robyn Littlewood, Simone Nalatu, Salifu Yusif and Jacqueline L. Walker in Journal of Primary Care & Community Health
