# Supplementary material for: Mixed Methods Evaluation of “Cooking Monsters”: An Empowerment-Focussed Aboriginal and Torres Strait Islander Adolescent Nutrition Programme
Source: J Prim Care Community Health. 2026 Feb 26;17:21501319261419935. doi: 10.1177/21501319261419935 (PMC12949285; doi:10.1177/21501319261419935)
Supplement: sj-docx-1-jpc-10.1177_21501319261419935 – Supplemental material for Mixed Methods Evaluation of “Cooking Monsters”: An Empowerment-Focussed Aboriginal and Torres Strait Islander Adolescent Nutrition Programme [file sj-docx-1-jpc-10.1177_21501319261419935.docx]

## Mixed Methods Evaluation of “Cooking Monsters” – an Empowerment-focussed Aboriginal and Torres Strait Islander Adolescent Nutrition Program - Supplemental Files

Supplement 1. Delivery of Cooking Monsters Program and Session Attendance

| Week | Program Activities | Attendance (n) |
| --- | --- | --- |
| 1 | Nutrition – Sugar Sweetened Drinks   - Sugary drinks education and discussion about diabetes and dental health. - Nutrition Information Panel reading. - Practical activity - sugar content in drinks. - Cooking Session – Healthy Smoothie. | 11 |
|  | Empowerment – Careers Planning Day   - Co-facilitated by a local tertiary education support service. - Pathways to higher education in rural towns. - Question and answer with community role models. - Adolescents identified potential career pathways and goals. | 11 |
| 2 | Nutrition – Iron and Anaemia   - Education and discussion about iron (deficiency, sources and absorption). - Cooking Session – Lentil Bolognese. | 16 |
| 3 | Empowerment – Story Mapping   - Story mapping (past, present and future) using creative drawing. - Identify inner strengths and positive attributes about self. - Program facilitators shared their own story mapping.   Empowerment – Team building   - Team building games to develop patience, communication and problem-solving skills. - Discussion about shame. | 13 |
|  | Nutrition – All about Vegetables   - Education and discussion about vegetables, chronic disease prevention and the function of nutrients. - Cooking Session – Chicken and vegetable curry. Calculated the serves of vegetables per serve of curry. Parents/caregivers were invited to taste the curry as part of a community barbeque and the health benefits of vegetables were discussed with the family unit. | 15 |
| 4 | Nutrition – Making Vegetables Yummy   - Discussion and education about how to cook vegetables to improve their taste/appeal. Education about the differences between frozen/fresh/canned vegetables and how to read nutrition labels of packaged vegetable products. - Adolescents (who had previously expressed strong disgust about vegetables) were asked to keep open mind about vegetables. - Practical activity – adolescents drew their usual dinner plate and compared it to a healthy plate model. - Cooking Session – healthy vegetables sides (e.g. honey/sesame carrots, zucchini/parmesan chips). - Home Challenge – Adolescents to take a photo of their dinner plate and match it to the healthy plate model. | 9 |
| 5 | Nutrition – Healthy Strong Bones   - Education about bone health and nutrition. Focus on eggs, their nutritional properties and the function of those nutrients. - Cooking Session – Omelette | 11 |
| Nil | All SEWB program activities cancelled for this week due to other events occurring within the local health service. | Nil |
| 6 | Empowerment – Preparation for Murri MasterChef   - Discussion about the competition, judges and prizes. - Menu planning/budget activity – Groups were required to develop a recipe and shopping list for their Murri MasterChef entry (within a predetermined budget). | 11 |
|  | Empowerment – Murri MasterChef Event   - Cooking session - Adolescents prepared their MasterChef entries independently with minimal input from facilitators. - The competition was judged by local community leaders, and there was a prize for the winner. - Adolescents prepared a short presentation to the MasterChef judges about their entry, with a focus on nutrition. - Distribution of Cooking Monsters Cookbooks. | 11 |

Supplement2 - Short Form Food Frequency Questionnaire Item Food Groupings for Analysis

| Food Group | Items Included |
| --- | --- |
| Fruit | Fresh fruit  Tinned fruit  Fruit juice |
| Vegetables | Salad  Vegetables |
| Total fruit/vegetables | Fresh fruit  Tinned fruit  Fruit juice  Salad  Vegetables |
| Whole-grain carbohydrates | Fibre rich breakfast cereal  Wholemeal bread |
| Discretionary snacks | Hot chips  Chips/savory snacks  Sweet biscuits, cakes, chocolate, sweets  Ice-cream/cream |
| Unprocessed meats | Beef, lamb, pork  Chicken, turkey  White fish not in batter, oily fish |
| Processed meats | Sausages, bacon, corned beef  Meat pies/pasties, burgers,  Chicken/turkey nuggets/twizzlers  Chicken pies, turkey burgers or in batter or breadcrumbs  White fish in batter or breadcrumbs |
| TOTAL Meat (Processed and unprocessed) | Beef, lamb,  Pork, chicken, turkey  White fish not in batter, oily fish  Sausages, bacon, corned beef  Meat pies/pasties, burgers,  Chicken/turkey nuggets/twizzlers  Chicken pies, turkey burgers or in batter or breadcrumbs  White fish in batter or breadcrumbs |
| Total serves fruit and vegetables | Daily serves of fruit  Daily serves of vegetables |

Supplement 3 - Nutrition Behaviour Pre- vs Post-Program Comparison for Individual Food/Drink Items (measured by a Food Frequency Questionnaire)

| Food/Drink Item | Pre-Program (n=15) | | Post-Program (n=8) | | Median of Pre- vs Post- Program Differences (n=8) (confidence interval)^***^ |
| --- | --- | --- | --- | --- | --- |
|  | Median | Interpretation | Median | Interpretation |  |
| Fruit (tinned or fresh) | 3 | Once per week | 3.5 | Between once and 2-3 times per week | 0 (1,7) |
| Fruit Juice | 4 | 2-3 times per week | 5 | 4-6 times per week | 0.5 (0, 4) |
| Salad | 2 | less than once per week | 1.5 | Between rarely/never and less than once per week | 0 (-2, 5) |
| Vegetables (cooked) | 4 | 2-3 times per week | 4^**^ | 2-3 times per week | 0 (-4, 2) |
| Hot chips | 5 | 4-6 times per week | 4 | 2-3 times per week | 0 (-4, 2) |
| Beans, pulses | 1 | Rarely or never | 3.5 | Between once and 2-3 times per week | 1 (-2, 3) |
| Fibre-rich breakfast cereals | 4 | 2-3 times per week | 2.5 | Between less than once and once per week | 0 (-5, 1) |
| Wholemeal bread | 3 | Once per week | 2 | less than once per week | -0.5 (-4, 1) |
| Cheese, yoghurt | 3.5^*^ | Between once and 2-3 times per week | 3 | Once per week | 0 (-6, 4) |
| Chips, savoury snacks | 5^*^ | 4-6 times per week | 3.5 | Between once per week and 2-3 times per week | 0 (-2, 3) |
| Sweet biscuits, cakes, chocolate, sweets | 4 | 2-3 times per week | 3.5 | Between once and 2-3 times per week | -1.5 (-3, 3) |
| Ice-cream, cream | 4^*^ | 2-3 times per week | 4 | 2-3 times per week | 1 (3, 4) |
| Sugar sweetened soft drink | 4.5^*^ | Between 2-3 and 4-6 times per week | 4 | 2-3 times per week | 0 (-2, 3) |
| Unprocessed red meat | 5 | 4-6 times per week | 4.5 | Between 2-3 and 4-6 times per week | -1 (-2, 1) |
| Unprocessed white meat | 5 | 4-6 times per week | 3.5 | Between once 2-3 times per week | -1 (-4, 0) |
| Processed red meat | 4 | 2-3 times per week | 3 | Once per week | 0 (-2, 0) |
| Processed white meat | 4^*^ | 2-3 times per week | 2.5 | Between less than once and once per week | -1 (-4, 3) |
| Processed fish (white) | 2 | Less than once per week | 1.5 | Between rarely/never and less than once per week | 0 (-3, 1) |
| Unprocessed fish (white) | 2 | Less than once per week | 1 | Rarely/never | 0 (-2, 0) |
| Oily fish | 1 | Rarely or never | 1 | Rarely/never | 0 (-2, 1) |
| Fruit | 1 serve per day^*^ | | 2 serves per day | | 0 (-1, 3) |
| Vegetables | 0.5 serves per day^*^ | | 0.75 serves per day | | 0 (-4, 2.5) |

^*^n=14 due to missing data

^**^n=7 due to missing data

^***^p>0.05

Supplement 4 - Additional Participant Quotes

| Themes and Key Messages | Participant Quotes |
| --- | --- |
| Theme 1: The Cooking Monsters Program is valued by community. | Adolescents  “It went amazing.”  “(It was good)… because um, it tastes good. Yeah, it looked good. Yeah. And it’s good for cooking. Yeah.”  “Oh the curry! The curry was good and so was the pasta.”  “It’s always fun”  “Cooking was Amazing. Yeah. Was Healthy!”  Researcher: “Good. Did you like it? All the cooking?” Adolescent: “We did and, mmhmm… Yeah. The um, the master chef.”  Adolescent: “Good. Yeah.” Adolescent: “Yeah.”  “And me and (Adolescent) liked the cooking.” |
|  | Staff  “I thought it went pretty good.”  “I think they’ve (adolescents) gained knowledge and enjoyed it. I think they’ve gained a bit of both actually.”  “But overall, I thought it was really good.” |
|  | Community Leader  “their peers involvement also was one of the greatest facilitator to those that did get involved and the shared sense of pride in their ability to prepare a meal for themselves and others.”  “It was great that there was a group of (Community Leader) that came it to do final judging for Murri MasterChef.”  “I think the program is something to be proud of. Whenever I have spoken to those outside of the community about the program they are always impressed that the community has these types of initiatives.” |
|  | Parent  “I think the program is a good thing for the community/ kids…”  “Yes, I would recommend it to others as it gives a sense of inclusiveness and education of how to cook and what to cook.”  “I think the program should keep going as it provides education and interest into future career fields”  “I was happy with my small experience as I got to taste some good food and it was great to see the excitement in the kids when they produced their food for us.” |
| Theme 2: Observable results included improved adolescent cooking and nutrition knowledge, and overall confidence and empowerment | Adolescents  Researcher: “What’d you learn?” Adolescent: “How to cook.”  Researcher:: “Yeah. Okay. Remember how I taught you how to read (sugar on the nutrition information panel)? Yeah. (pointing to a snack item).” Adolescent: (Reading nutrition label) “So, um, sugar and in the last one is 24.4”.  Researcher: “Yeah. Is that per serve or per hundred? Which one are you meant to be looking at?” Adolescent: “Per hundred. Yeah.” Researcher: “Cool. And when it’s a drink, how much are you aiming for?” Adolescent: “Zero.”  Researcher: “So did you feel like you learned stuff?”  Adolescent: “Mm-Hmm”  Researcher: “What about the cook book? What about the other stuff we talked about? Like the sugars?” Adolescent: “And… Oh, how it was good for you (referring to the cookbook)? Mm. The healthy ingredients that go in it. Yep.”  Researcher: “So do you guys feel like that was good to know and you remembered it?” Adolescent: “Mm-Hmm”  Adolescent: “Oh, like the foods? Yeah. How we learn how to like chop up stuff and be careful (in the) kitchen. Be careful with the knife”  Researcher: “Do you think learning how to cook helped with your healthy mind or it didn’t make much of a difference?” Adolescent: “Yeah.”  Researcher: “Like it built your confidence?” Adolescent: “Maybe”  Adolescent: “Yeah”  Adolescent: “Yeah”  Researcher: “What about you (name of adolescent), did you feel cooking, build your confidence? Have you cooked any of them at home?” Adolescent: “Me, um, you know, I made spaghetti Bolognese (at home) but with mince.” |
|  | Staff  “And I, and just from that… I was iffy about how much knowledge they’d gained. Mm-Hmm. Um, because it was only one afternoon a week… Um, but watching that, you could tell that they were listening because they did gain some knowledge. I think their shyness sometimes doesn’t express… doesn’t Let them express how much they have gained.”  “Uh, I, I, I think we’ve seen, well I have seen the kids grow from the start of the year Mm-Hmm. Until now. Mm-Hmm. Um, it, it’s a, it’s a tricky one because some of the games that you, that we target just to build a bit of confidence and self-esteem and empowerment and stuff like that, they don’t really know that that’s what you’re doing. Yeah. They think it’s just a game. Yeah. Um, which is good.” |
|  | Community Leader  “Yes, I believe that teaching our young people important life skills is a massive benefit that will empower their decision making and lifestyle choices into the future. I think there has been a change as kids have been getting excited to be able to prepare these meals or even talk about meals they are going to cook away from the program. We attended a dinner event for the SEWB Program group and (local university) Students and the kids were proud to present their dinner to the wider group and it was the first to completely disappear.” |
|  | Parent  “Yes, it was beneficial to the kids as it gave them the opportunity to cook their own food and also learn about the food they were cooking.”  “Some of the kids are talking about cooking at home.” |
| Key Message 1: The program increased in its acceptability over time. | Staff  “Yeah, totally. Because they’ve been at school all day learning when they get to here, they wanna have some fun and do some um Yeah. Physical stuff. Yeah.”  “..I was surprise, I was surprised how much they enjoyed it. Um, I didn’t think they would. “  “uh, ’cause when, when I was talking to ’em before we come back about, you know, we’re gonna be doing some cooking, they were like, ‘oh’. Like, I think they (adolescents) thought it was gonna take away from what they do with (the SEWB program). Yeah. Um, but you’ve seen on Murri Master Chef Day. Mm. How they, how much they were into it and how much pride they had in what they were doing. So much pride. Yeah. And, um, and they wanted to know the knowledge and they wanted to be able to. Yeah. Make… Have the food skill now ’cause they wanted to impress everyone. Yeah. And, and they did. It was all nice. They did good.”  “There is good potential for it to run some good stuff and to do some good stuff with them in this, because I was sceptical at the start. I was like, I don’t know if these kids are wanting to cook, I think now that they’ve done it, and they got into it.”  “They play games here. Some don’t want to play that game, so. That’s not near here nor there. But I think now that they’re understanding and what you’re doing, we can fix a few little things up like that. They’ll be a hit and hit a runner.” |
|  | Community Leader  “Also being able to make the activities enjoyable and fun (increases program acceptability).” |
| Key Message 2: The pilot was viewed as a successful learning opportunity, during which, recommendations that would improve the program were identified. | Staff  “Mm. Um, obviously everything that we do, whether it’s (SEWB Program) or whether it’s your cooking stuff, there’s, there’s always room for, you know, a little bit of, I think it was improvement ’cause it’s just a trial. Yeah. Everything we do at (SEWB Program) is just the trial. Yeah. See how it goes. And reflect… Pretty much what we’re doing. Yeah. Try and better with something else that we do next time.”  “So that sort of stuff. Yeah. Um hmm. But it was good. It is like, like you said, it’s a trial one “  “Yeah. Well, like, it’s a lot of stuff we do. Like, you know, even the (SEWB Program activities), it’s trial and error”  “There’s lots of stuff that we can fix up there and do there differently and, but it’s work in progress.”  “It’d be good like, you know, I don’t know whether it’s whatever, how many months, but go back, go through all that. Have a look how it all went. Yeah. Take some of the suggestions that we’ve both spoke about and then come back. Yeah. And file it into ’em. Like, and go, righto, this is where what we’ve identified out of that. Yeah. Um, let’s go back again and really… Yeah. Really get it done and see where it takes us this time. Yeah.”  “But I think it would be good to come back again and rerun the programme Yeah. With a couple of, um, you know, of the suggestions we made.”  “No, like I said, it was good. And it was probably a learning thing for me too.” |
| Key Message 3: Further embedding Cooking Monsters within the local implementation environment could increase its degree of fit and improve engagement. | Adolescents  “But no one (out of the boys who preferred playing games) wanted to do cooking. They wanted to do games.” |
|  | Staff  “I think one of the ways we could probably do that (improve planning through further program integration) is, you know, on the Monday you’d come in and say, ‘righto, this week we’re cooking it’s spag bowls, some Bolognese. Here are the ingredients and this is how we cook it,’ like doing (demonstration). And then on the Wednesday, um, you actually cook it where, you know, we might send five into you first up, while I’m doing stuff with five, then swap ’em over so they all know what they’re doing. Yeah. They’ve all got the, um, idea because we’ve spoken to ’em on Monday that that’s what’s going to happen. These are ingredients, this is how you cook it. Yeah. And then those five go through and actually do it. Then the next five then, so they all know what they’re up to. They all know what they’re cooking and away we go.”  “It (Cooking Monsters) could be incorporated to the point system… Um, I’m just thinking of, of a couple of boys that had no interest in it. Mm. Um, I think it, I think also, if it was something that was consistently put into the plan. Mm. Consistently week in, week out, um, I think they, that those guys would start to enjoy it. Especially if they, you know, like when we’re making our stuff for, um, for afternoon tea today, like Yeah.”    “Because I think if you come back and go, righto, this is how we’re gonna run the cooking. This is what we’re gonna target first. This is… do your lesson plan up. This is the first one, this is the second one. This is whatever. The first one will be about the safety in there (the kitchen). And you know, you can play little games around that. Yeah. And then get into the cooking and get into the… ’cause they know they, they got a little bit of understanding what it’s about now. So I think the next one, if you, you know, on the days that you’re not doing the cooking, get in and do stuff with them. Mm mm They’ll, um, I think you’ll capture better evidence and measures next time.”  “Yeah. And I.. I also think like if you can get those guys that have no interest in it to actually cook something that they really like and they want to cook it. Yeah. And then they’re like very proud of it. Um, I think they’re inclined to do it again. Yeah. Um, yeah. That is a tricky one. Mm. I suppose if we had the answer to that, Then we’d have the answer and teaching would be easy at school.”  “Yes. Because they just stay down there (playing games and not engaging in the program).”  “There’s a few there that didn’t want to be part of it, but then you’re going to get that with anything.” |
|  | Parent  “It is something that could be moulded into other activities.”  “I would like to see it moulded into some of the other activities as well. There is an opportunity that something similar could be included (other SEWB program activities)” |
| Key Message 4: Overcoming resource, time and equipment constraints could improve program delivery in the future. | Adolescents  “I feel like there shouldn’t be a lot of people in the kitchen at once. Like last time.” |
|  | Staff  “Um, probably equipment probably, um, you know, would be hard (there is not enough). I suppose if (the health service) or, or the kitchen had four fry pans and Yeah. Things like that would… (help).”  “Instead of doing a five week or… however long it went for. Mm. It’s eight weeks so that they’re actually, um, you know, we’ll get something out of it and maybe take home. I think the shorter one was just like an introduction for ’em. Yeah. Whereas I don’t know that they’ll go home now and, and they, they may well do, but I think, um, a longer one where get into a routine. Um, yeah. Will be.. will be beneficial for ’em.”  Researcher: “… Something that I was most challenged by, I think, is trying to get all of them through to cook something in a short amount of time. “ Staff: “Yes. When you’ve got lots of kids and they’re all sort of running around crazy.”  “Yeah. I think, um, with, with the one that you’ve done, I, I think just having, having more time with you. Two days a week over an eight week thing. Yeah. Or one day a week over a 10 week.”  “Um, so I, I don’t know, but I’m just trying to work out a way how you can get through 20 kids or 25 kids or whatever it is, that are in an activity through cooking. Whether we split ’em into different groups, whether it’s five and this group’s cooking this on Monday, this one’s cooking this on Tuesday so that all the others are occupied doing something, then you’ve just got five kids in there doing it. Yeah. I don’t know.”  “Yeah. And whether you do it in groups, whether it’s a group of two or a group of three and they’re all making the whatever it is that they’re cooking. Yeah. And if (the health service) have a couple, um, gas burners with, with whatever they need, and you can put tables out in this big area to cook.“  “But (the health service) need to have their own of all these particular (cooking) things. Yeah. And you can, we’d set the tables out here and do the cooking demonstration with them. Mm. Um, yeah. Yeah. I think that like a lot of little stuff like, like, like that there’s (the health service) having all their own equipment to make it, um, functionable for… To do big group cooking.  “So I don’t know, like, there’s lots of things. It’s just, um, the tricky thing is, like you said, it’s the amount of kids, um, having all the equipment that’s needed to make it a successful so that every kid can, every kid can cook or do it in groups. Yeah. Um, yeah.”  “And just the time in the afternoon… Um, you know, by the time we get here, that’s half past three, depending how many drop offs we do. It could be quarter to four. So you’ve pretty much then got an hour… Yeah. So, um, whether it’s you prep it up and then you’re here through the school holidays where you can, you know, the two week walkthrough of school holidays, you can really drum it up. Oh yeah. Because you’ve got ’em all day. Yeah. So, whether that’s better?”  “Um, if you come out, you know, a couple of weeks, four weeks or whatever before the school holidays started prepping, uh, one day a week or a couple days a week and then school holidays. Yeah. It’s a big thing, they could cook their lunch, they could cook their dinner.”  “Um, you know, the ideal would be too, is to take ’em shopping, Yeah. And buy the groceries. Yeah… But again, again, it comes down to having 20 kids going shopping with you. Yeah. Um, and how, how you break that up. Yeah. And there’s only like, you know, even if it was yourself and me as the coordinator, you, you probably still need another one… Yeah. It’d be hard even if we did that like, I don’t know how you do it, because they need to be able to go with yourself being the dietitian. So you can talk about all the labels, all the nutrition and all that of the food.” |
|  | Parents  “It (the program) needs more time and effort of those that are involved spending time with the kids.”  “I do think being happy with program helps a little bit but it does need more than that. It needs interest and backing from organisations and time and effort from people who are involved.” |
| Key Message 5: Relationships are central to program success and need to be continued on-going. | Staff  “You really have to build a relationship and for you to build a relationship, you’ve gotta be around a little bit. Mm. And participating in everything... that they’re doing. Mm-Hmm.”  “Mm. And I think once you gain that and gain their respect, I think you’ll be able to get them to do most things.”  “So I think the next one, if you, you know, on the days that you’re not doing the cooking, get in and do stuff with them. Mm mm… They’ll, um, I think you’ll capture better evidence and measures next time.”  “Someone new coming out, they’re going to not do too much for the first month or whatever. It’ll be just about getting there, forming a (relationship).” |
|  | Community Leader  “Of high importance is their (adolescents) relationship with the SEWB program Coordinator. This person is key to increasing willingness to participate in these programs and help connect the dots between their experience and life lessons which is empowering.”  “The sense of ownership will definitely help the programs sustainability as there are multiple stakeholders that now would have seen the success and would like that to keep going. A big part of this is reliant of the Big Buddy Coordinator continuing this type of project in a way that the kids find enjoyable.” |
